# Choroidal pericytes promote subretinal fibrosis after experimental photocoagulation

**DOI:** 10.1242/dmm.032060

**Published:** 2018-04-23

**Authors:** Xueting Luo, Shiqi Yang, Jian Liang, Yuanqi Zhai, Mengxi Shen, Junran Sun, Yiji Feng, Xinmin Lu, Hong Zhu, Fenghua Wang, Xiaodong Sun

**Affiliations:** 1 Department of Ophthalmology, Shanghai General Hospital (Shanghai First People's Hospital), Shanghai Jiao Tong University School of Medicine, 100 Haining Road, Shanghai 200080, China; 2 Shanghai Key Laboratory of Fundus Disease, Shanghai, China; 3 Shanghai Engineering Center for Visual Science and Photomedicine, Shanghai General Hospital (Shanghai First People's Hospital), Shanghai Jiao Tong University School of Medicine, 100 Haining Road, Shanghai 200080, China

**Keywords:** Age-related macular degeneration, Fibrosis, Pericyte

## Abstract

Subretinal fibrosis results in local destruction of retinal structures and permanent vision loss, representing the end stage of neovascular age-related macular degeneration (AMD). Histological examination of fibrotic specimens from AMD patients has uncovered a wide range of cellular and acellular components. However, their origins and roles in fibrosis remain largely unexplored. Using a laser-induced photocoagulation model with collagen 1α1-GFP reporter mice, we demonstrate, by cell-lineage tracing, that pericytes associating with choroidal microvasculature are activated upon injury and infiltrate into the subretinal space as significant components of fibrotic lesions. In contrast to their choroidal precursors, infiltrating pericytes acquire stellate-like structures, upregulate expression of fibrogenic molecules and colocalize with extracellular fibrotic scar. Collectively, our results identify the choroidal perivascular niche as a novel source of subretinal fibrosis after photocoagulation, and suggest that collagen 1-expressing pericytes are potential targets for therapeutic intervention to suppress subretinal fibrosis and preserve vision.

## Abstract (precis)

**Summary:** Using a laser-induced photocoagulation model with collagen 1α1-GFP reporter mice, we identified choroidal pericytes as potential therapeutic targets to prevent subretinal fibrosis for management of neovascular age-related macular degeneration.

## INTRODUCTION

AMD is a leading cause of low vision and blindness in industrialized countries. There are ∼30 million AMD patients worldwide and the prevalence increases with age, affecting 25% of the elderly. At advanced stage, 10-15% of AMD patients develop choroidal neovascularization (CNV) and undergo rapid progression to severe vision loss if left untreated (Ambati and Fowler, 2012; Lim et al., 2012). Anti-vascular endothelial growth factor A (VEGF) therapy is the first-line treatment for CNV, and has achieved great success in suppressing pathological angiogenesis and rescuing vision. However, the beneficial effect of anti-VEGF therapy diminishes, with the visual function decreasing to baseline level within years (Rofagha et al., 2013). Moreover, a substantial portion of CNV patients showed poor response to anti-VEGF therapy (Cohen et al., 2012), suggesting nonangiogenic mechanisms that propel disease progression.

Subretinal fibrosis develops in concurrence with CNV even in the presence of anti-VEGF therapy (Daniel et al., 2014). It features complex interaction between cellular components within subretinal lesions and local inflammatory factors, which ultimately leads to remodeling of the extracellular matrix (ECM) and subretinal scarring. Subretinal fibrotic scars exacerbate the local microenvironment and destruct the visual axis (Ishikawa et al., 2016). Thus, subretinal fibrosis represents an obstacle to neovascular AMD management. However, owing to complexity of the fibrotic processes, effective therapeutic intervention for subretinal fibrosis has not yet been developed. Although previous reports have identified some cellular components of subretinal lesions (Grossniklaus and Green, 1998; Grossniklaus et al., 1994; Lopez et al., 1996), the lack of a lineage tracing system hinders further identification of the origin and the role of participating cells during subretinal fibrosis.

Collagens are the major ECM deposits that constitute fibrotic scars (Das et al., 1992; Ishikawa et al., 2016), and, consistently, collagen-producing pericytes have been shown to participate in tissue fibrosis in liver, kidney and spinal cord after injury (Giuliano and Pagès, 2013; Soderblom et al., 2013; Yata et al., 2003). Therefore, we reason that collagen-producing cells might participate in the pathogenesis of subretinal fibrosis. In this study, we employed transgenic mice in which GFP expression is driven by collagen type 1 promoter (Col1α1-GFP mice) (Yata et al., 2003). We show that, in Col1α1-GFP mice, a subset of pericytes in the choroidal vasculature is labeled by GFP, which was taken advantage of as a lineage tracer to study the perictyes’ role in subretinal fibrosis. We describe that, after photocoagulation, a robust distribution of choroid-derived pericytes constituted the fibrotic lesions, with upregulated expression of ECM components and demarcated territories of ECM deposits. Collectively, our data point to the choroidal perivascular niche as a novel and significant source of fibrotic lesions after photocoagulation and a potential therapeutic target for intervention of subretinal fibrosis.

## RESULTS

### GFP labels a subset of choroidal pericytes in Col1α1-GFP mice

**Fig. 1.**
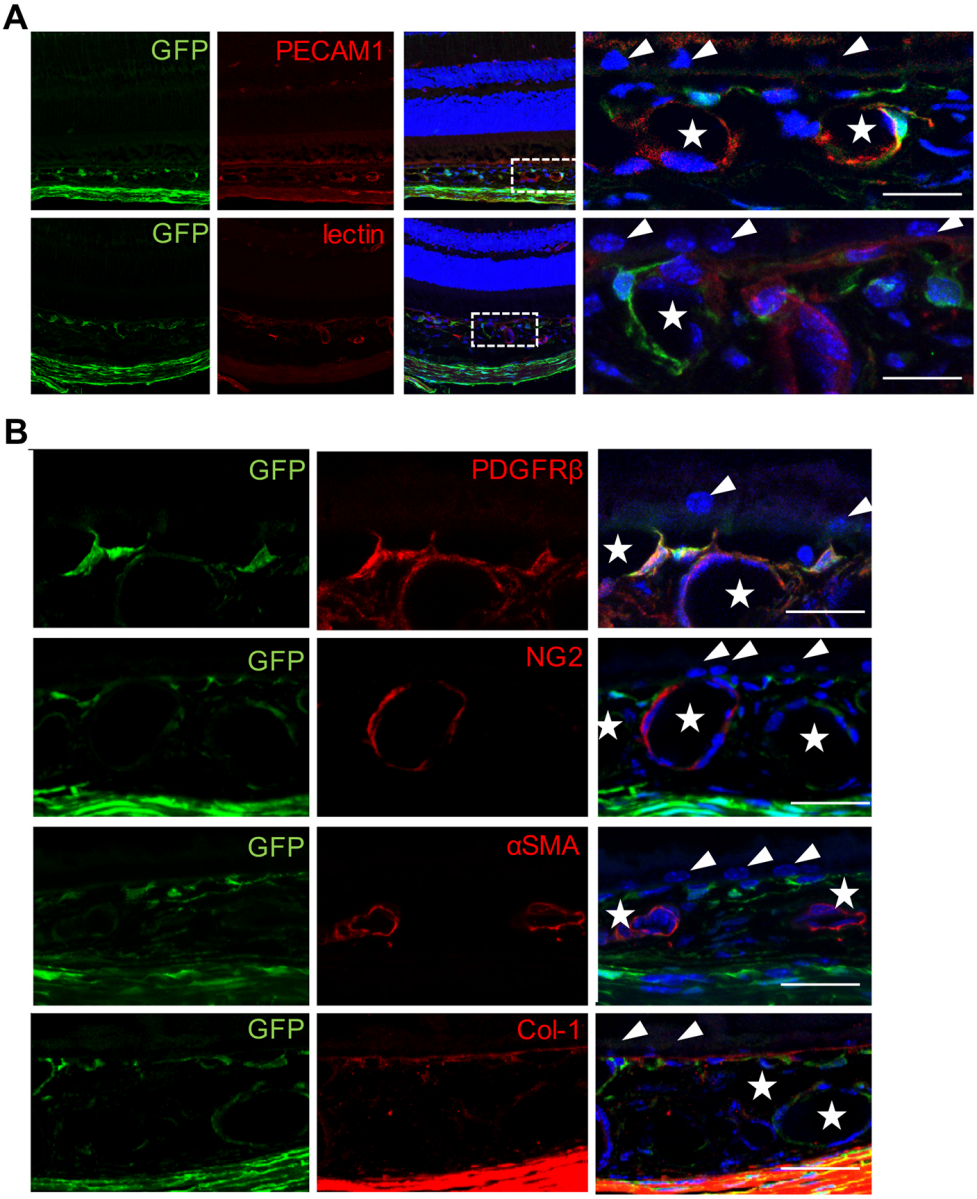
**GFP labels choroidal pericytes in Col1α1-GFP mice.** (A) GFP-positive cells preferentially associate with choroidal microvasculature beneath the RPE layer without staining with fluorophore-conjugated lectin or anti-PECAM1 antibodies (*n*=5). The boxed areas are shown at higher magnification on the right. Stars indicate the choroidal microvasculature; arrowheads indicate RPE cells. (B) GFP-positive cells express the pericyte marker PDGFRβ but not NG2, αSMA or collagen 1 (*n*=5). Samples were stained with DAPI (blue), GFP (green) and the indicated antibodies (red). Scale bars: 20 μm.

To localize GFP-positive cells in the choroid, we performed immunohistochemistry on cross sections of the posterior pole of eyes. GFP-positive cells appeared to juxtapose the microvasculature or wrap with their processes around microvessels beneath the retinal pigment epithelial cell (RPE) layer. GFP-positive cells did not express endothelial marker PECAM1 and were not labeled with fluorophore-conjugated lectin (Fig. 1A). Although antibody against collagen 1 labeled Bruch's membrane, it did not appear to label GFP-positive cells in the choroid (Fig. 1B). To further identify the cellular identity of choroidal GFP-positive cells, we performed antigenic profiling analysis with a comprehensive list of cellular markers, as listed in Table 1. Most GFP-positive cells expressed PDGFRβ (92.8%), a marker commonly used to identify perivascular cells, but did not appear to be positive for NG2 (Cspg4), another pericyte marker (Fig. 1B, Table 1). In addition, antibody against αSMA (Acta2) marked vascular smooth muscle cells around microvessels in the choroid other than the GFP-positive cells (Fig. 1B, Table 1). We also analyzed cellular markers for macrophages/microglia (Iba1; Aif1), fibroblasts (CD13; Anpep), glia (GFAP) or RPEs (RPE65), but found no overlap with GFP signals (Table 1; Fig. S1). Therefore, based on the distribution and antigenic profile, we conclude that GFP-positive cells in the choroid are predominantly pericytes (GFP-positive pericytes).

**Table 1.**
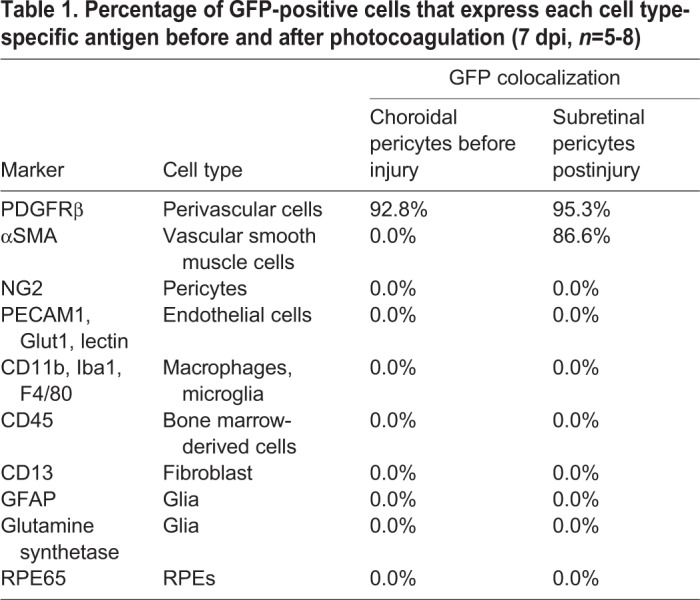
**Percentage of GFP-positive cells that express each cell type-specific antigen before and after photocoagulation (7 dpi, *n*=5-8)**

### GFP-positive pericytes constitute a significant component of subretinal lesions after laser-induced photocoagulation

**Fig. 2.**
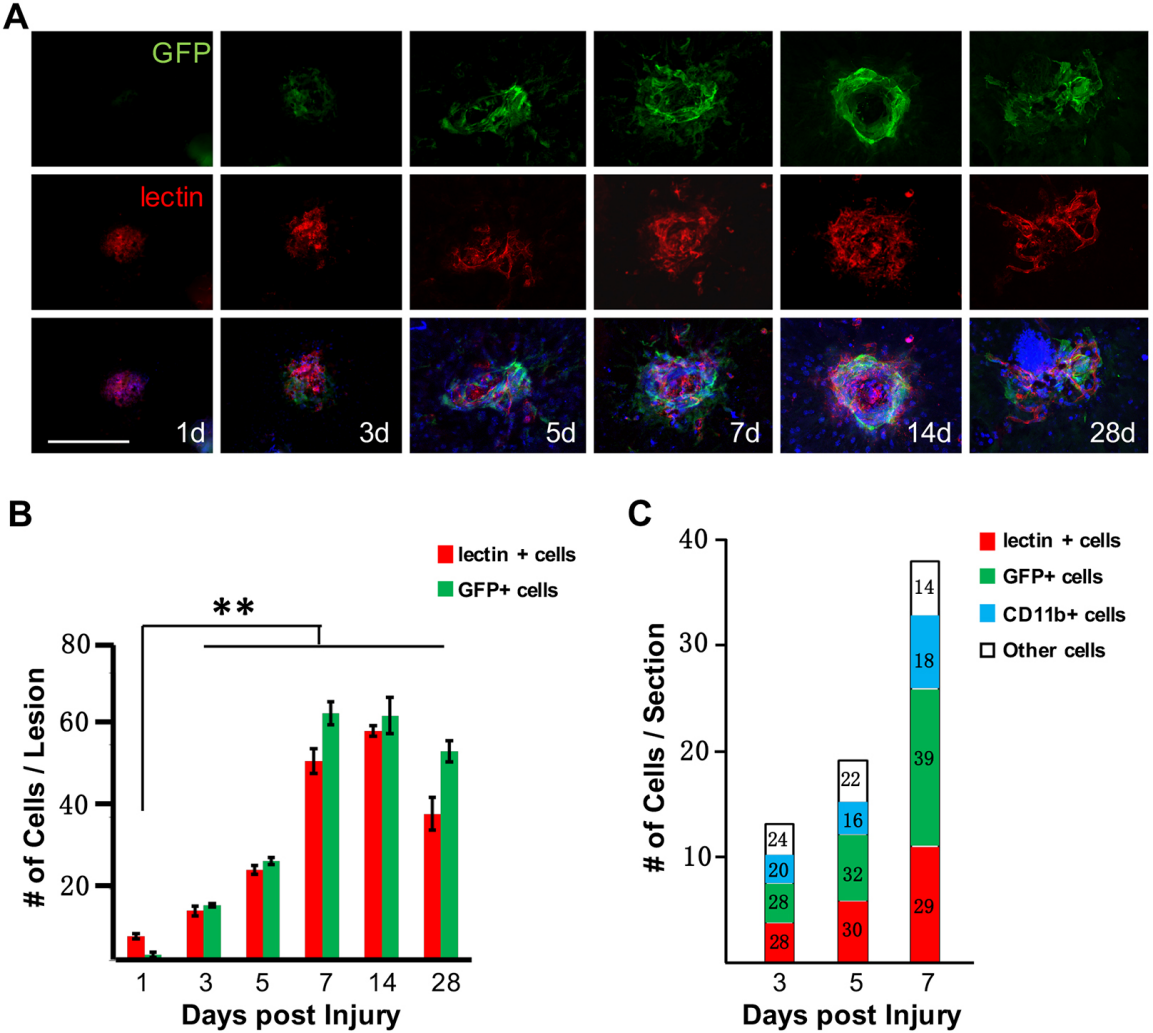
**GFP-positive cells are significant components of laser-induced lesions.** (A,B) The time course of GFP-positive cell distribution in the subretinal space after laser-induced photocoagulation (*n*=8). Scale bar: 100 μm. (C) Cell count analysis of lesion epicenters (numbers in the bars designate percentages, *n*=6). **P<0.01 (one-way ANOVA).

To investigate the contribution of GFP-positive pericytes to the development of subretinal lesion characteristic of neovascular AMD patients, we performed laser-induced photocoagulation in Col1α1-GFP mice. In this model, the fibrotic tissues persist for over 35 days after laser treatment, mimicking the natural pathogenic course of patients with neovascular AMD who develop fibrotic scarring subsequent to CNV (Daniel et al., 2014; Ishikawa et al., 2016). We analyzed flat-mounted RPE-choroid complexes at multiple time points after injury. One day postinjury (dpi), vascular endothelial cells infiltrated into the subretinal space, reaching a peak on 7 dpi (Fig. 2A,B), consistent with previous findings (Ishikawa et al., 2016). On the other hand, GFP-positive cells were not detectable in the subretinal space until 3 dpi. The number of subretinal GFP-positive cells continued to increase and also peaked on 7 dpi (Fig. 2A,B). We noticed that the subretinal GFP-positive cells closely associated with, but did not overlap with, lectin-labeled endothelia at all time points tested (Fig. 2A), indicating their nonendothelial origin. Moreover, these GFP-positive cells appeared to elongate progressively and surround the lesion after 7 dpi. Thereafter, both the GFP-positive cells and endothelia regressed on 28 dpi (Fig. 2A,B).

The structures of GFP-positive cells in the lesion sites are reminiscent of the ‘myofibroblastic scaffold’ composed of PDGFRβ^+^/αSMA^+^ cells as previously reported (Strittmatter et al., 2016). To further determine the cellular identity of infiltrating GFP-positive cells, we stained the flat-mounted laser-injured RPE-choroid complexes with antibodies against PDGFRβ and αSMA. Interestingly, most GFP-positive cells expressed PDGFRβ and αSMA markers (Table 1; Fig. S2), indicating that choroid-derived pericytes are contributors to the ‘myofibroblastic scaffold’.

In order to quantify the contribution of GFP-positive cells to subretinal lesion, we obtained consecutive cross sections of the lesion sites on 3, 5 and 7 dpi and chose the lesion epicenters for cell count analysis. As shown in Fig. 2C, the total number of infiltrating cells within lesions increased from ∼13.4 per section on 3 dpi to ∼37.1 per section on 7 dpi, with relatively stable contribution of endothelia and microglia. In sharp contrast, the percentage of GFP-positive cells increased from ∼28% on 3 dpi to ∼39% on 7 dpi. These results strongly support a significant role of the GFP-positive cells to the development of subretinal lesions after laser-induced photocoagulation.

### Subretinal GFP-positive cells originate from choroidal pericytes

**Fig. 3.**
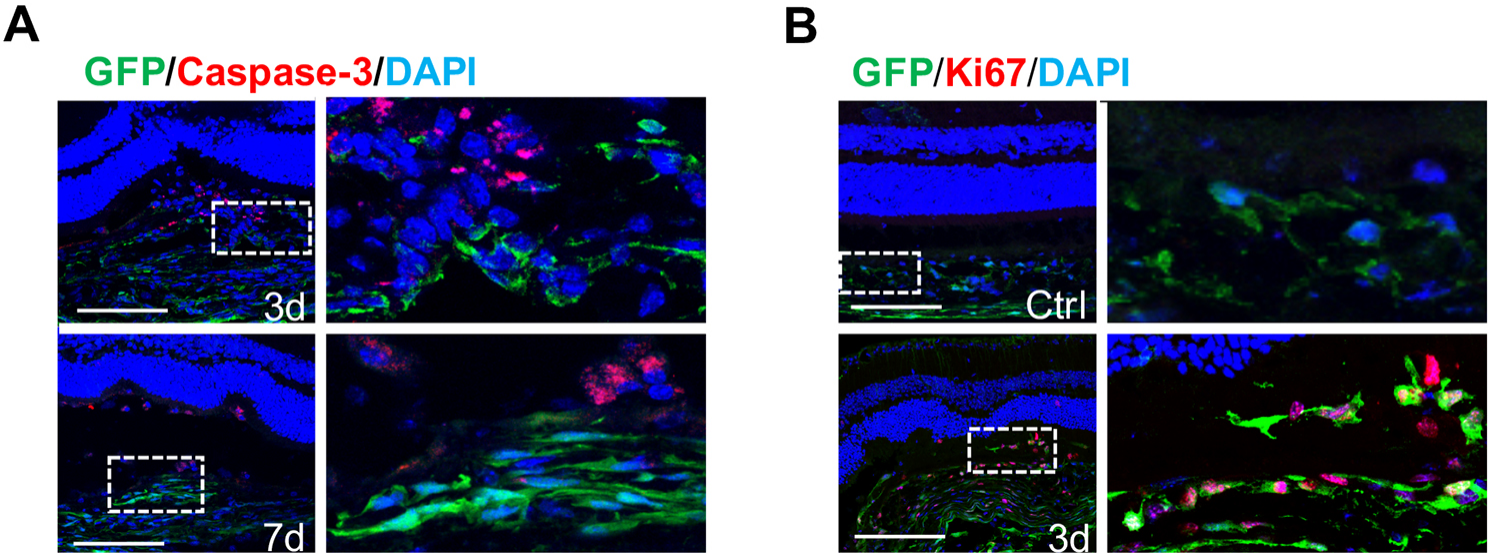
**GFP-positive pericytes around the lesions are activated after laser-induced photocoagulation.** (A) Caspase 3 signals were detected around the lesions at 3 and 7 dpi but were not associated with the GFP-positive cells (*n*=6). (B) Ki67 signals are detected in the GFP-positive pericytes around the lesions at 3 dpi (*n*=6). Scale bars: 100 μm.

Owing to the close relationship between choroidal pericytes and microvasculature where neovascularization derives, we speculate that the GFP-positive cells, as seen in the subretinal lesions, could also originate from the choroid. We analyzed cross sections of subretinal lesions on 3 and 7 dpi. As shown in Fig. 3, the choroidal structures were disrupted after laser injury. Trails of GFP-positive cells appeared to activate by upregulating GFP expression levels and filled the RPE breach. Apoptotic cells were readily detected around the lesion sites on 3 and 7 dpi with perinuclear staining of caspase 3 signals (Fig. 3A). We carefully went through consecutive optical sections taken with confocal microscopy to trace GFP and caspase 3 signals. In general, the anti-caspase 3 antibody did not stain infiltrating GFP-positive cells. We noticed a significant increase in subretinal GFP-positive cells from 3 to 7 dpi (Fig. 2A,B). One explanation is *in situ* proliferation of GFP-positive pericytes in the choroid after injury. Indeed, we detected robust proliferative signals in the GFP-positive cells around the lesions on 3 dpi (Fig. 3B). The percentage of GFP-positive cells with Ki67 (Mki67) signals around the lesion sites was determined as 62.6±12.5%.

**Fig. 4.**
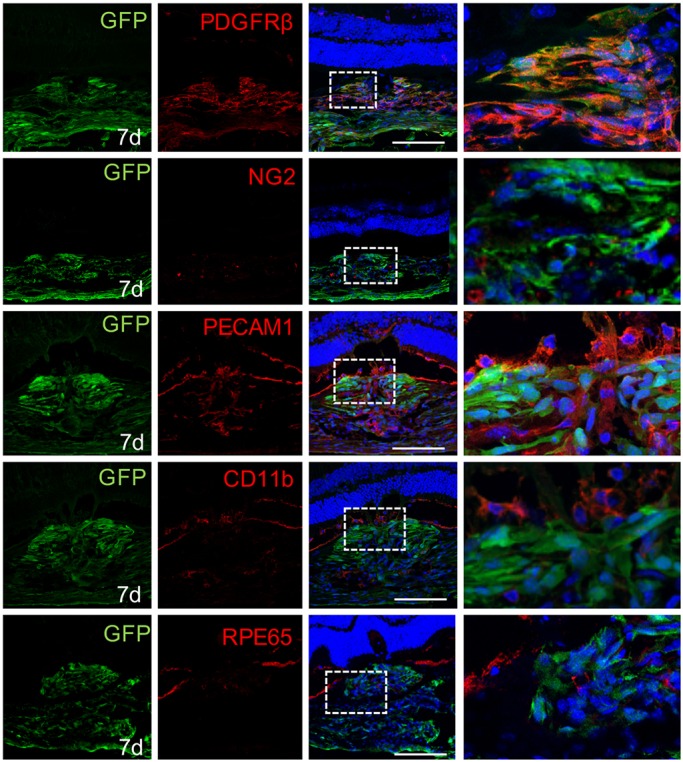
**Subretinal GFP-positive cells express pericyte markers.** GFP-positive cells within the lesions express PDGFRβ, but not NG2, PECAM1, CD11b or RPE65 (*n*=8). Scale bars: 100 μm.

However, at this point, we could not rule out the possibility that cells other than GFP-positive pericytes are triggered upon injury to express GFP. Therefore, we set out to determine the cellular identity of subretinal GFP-positive cells by antigenic profiling. We performed immunohistochemistry on cross sections of subretinal lesions at 7 dpi using the same list of antibodies as described above (Table 1). Most GFP-positive cells (95.3%) at the lesion site were co-labeled with anti-PDGFRβ antibodies (Fig. 4, Table 1), indicating their perivascular origin. We also tested antigenic markers for pericyte (NG2), endothelia [PECAM1 and Glut1 (Slc2a1)] and macrophage/microglia [CD11b (Itgam) and Iba1], but, without exception, none of the GFP-positive cells were labeled (Fig. 4, Table 1; Fig. S3). Previous reports suggest that shed RPEs contribute to subretinal lesion through epithelial-mesenchymal transition (Grisanti and Guidry, 1995; Lopez et al., 1996). Although antibody against RPE65 specifically labeled RPEs around or within the lesion site, no signal was detected in the GFP-positive cells on 7 dpi (Fig. 4). Owing to the elongated morphology of subretinal GFP-positive cells on 7 dpi, we speculate that these could be fibroblasts which have been recognized as crucial components of fibrotic tissues (Hinz and Gabbiani, 2010; Wynn and Ramalingam, 2012). Vimentin and CD13, markers frequently used to label fibroblasts, were abundantly expressed in the lesion sites but neither was detected in the GFP-positive cells (Table 1; Fig. S3). Breakdown of the blood-retina barrier is a feature of AMD pathogenesis and occurs in experimental photocoagulation (Ambati et al., 2013). As a result, bone marrow-derived circulating cells gain access to subretinal lesions (Espinosa-Heidmann et al., 2005). Herein, we stained for CD45 (Ptprc)-positive bone marrow-derived cells at subretinal lesion sites. Although clear CD45 signals were detected within the lesions, they did not overlap with GFP-positive cells (Fig. S3). In addition, glial markers GFAP or glutamine synthetase did not label the subretinal GFP-positive cells (Fig. S3).

In summary, the infiltrating GFP-positive cells expressed pericyte-specific markers PDGFRβ, but not NG2 or markers for endothelial cells, macrophages/microglia, fibroblasts, RPEs, bone marrow-derived cells or glia.

### Subretinal GFP-positive cells are sources of ECM

**Fig. 5.**
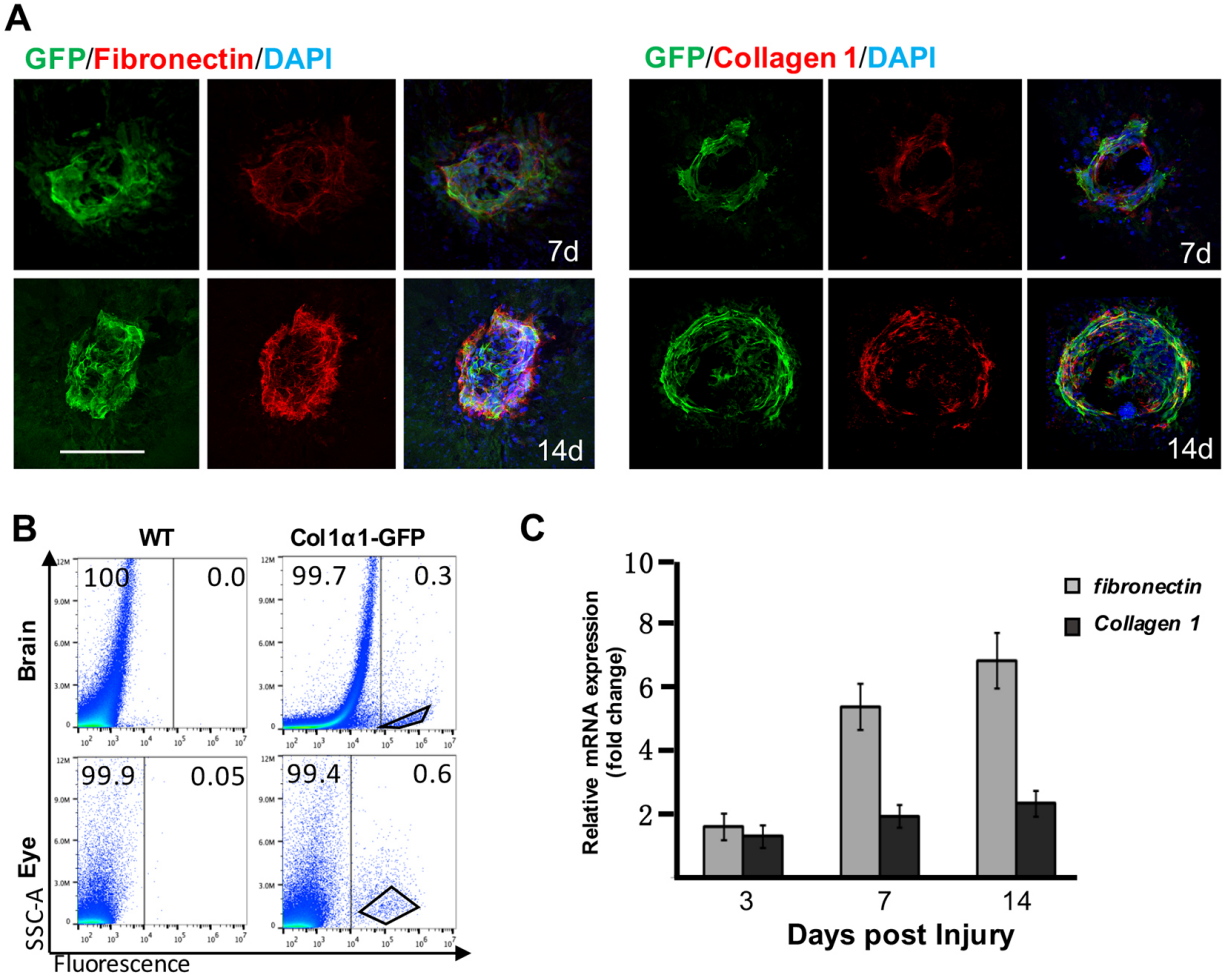
**Subretinal GFP-positive pericytes express and demarcate territories of fibronectin and collagen 1.** (A) Distribution of fibronectin and collagen 1 was constricted by GFP-positive pericytes in the subretinal space (*n*=5). Scale bar: 100 μm. (B) GFP-positive pericytes were isolated by FACS for quantitative PCR analysis. (C) Relative expression of collagen 1 and fibronectin mRNA from FACS-isolated cells was normalized to that of *Gapdh*.

Excessive deposition of ECM is characteristic of tissue fibrosis that ultimately develops into scars. Previous studies with clinical samples have identified fibronectins and collagens as components of subretinal fibrotic lesions in AMD patients (Das et al., 1992; Ishikawa et al., 2016). Interestingly, infiltrating GFP-positive cells expressed myofibroblastic marker αSMA (Table 1; Fig. S2). Therefore, we speculate that these infiltrating GFP-positive pericytes are potential sources of ECM. We stained flat-mounted RPE-choroid complexes on 7 and 14 dpi with antibodies against fibronectin and collagen 1, the major components of ECM deposits. Interestingly, the expression pattern of fibronectin and collagen 1 overlapped to a large degree within the territories of infiltrating GFP-positive pericytes (Fig. 5A). To test whether infiltrating GFP-positive pericytes express ECM components, we employed fluorescence-activated cell sorting (FACS) to isolate GFP-positive pericytes in the subretinal space on 7 dpi for transcriptional analysis. GFP-positive pericytes in the choroid are difficult to purify, owing to enrichment of GFP-positive fibroblasts in the sclera (data not shown). In return, we purified the GFP-positive pericytes from the cortex to serve as a reference (Fig. 5B). Of note, subretinal GFP-positive pericytes upregulated transcription of ECM components (i.e. collagen 1 and fibronectin) from 3 to 7 dpi (Fig. 5C). We also compared the expression pattern of fibronectin with the distribution of other cell types (i.e. endothelia, microglia and bone marrow-derived cells) by double staining, but no obvious correlation was found (Fig. S4). Based on these histologic and molecular analyses, we conclude that infiltrating GFP-positive pericytes are major contributors to ECM deposits after laser-induced photocoagulation.

## DISCUSSION

Subretinal fibrosis represents the end stage of neovascular AMD and has been considered as a significant risk factor for vision impairment (Ishikawa et al., 2016; Lim et al., 2012). However, the mechanism of subretinal fibrosis remains largely unexplored (Friedlander, 2007). Our data obtained using genetic fate-mapping techniques suggest that after photocoagulation, a subset of choroidal pericytes infiltrate into the subretinal space, proliferate and contribute to neovascular lesions as significant components. Subretinal pericytes express and demarcate distribution of ECM components, which indicates a crucial role of choroidal pericytes in subretinal fibrosis as occurred in end-stage AMD. Our findings suggest that choroidal pericytes could be potential therapeutic targets for suppressing subretinal fibrosis.

Previous studies indicated a transition of RPEs from quiescent (vimentin^+^ and αSMA^−^) to activated myofibroblast-like (vimentin^−^ and αSMA^+^) phenotypes by histological analysis of clinical specimens (Lopez et al., 1996). Moreover, cultured RPEs can be induced to undergo epithelial-mesenchymal transition and acquire myofibroblast-like phenotypes, as determined by expression of αSMA and ECM components (Grisanti and Guidry, 1995). Therefore, it is widely thought that RPEs are the primary source of subretinal myofibroblasts that promote fibrosis. However, definitive evidence for transdifferentiation from RPEs to myofibroblast *in vivo* is not available. In this study, we show that infiltrating GFP-positive cells acquired myofibroblastic phenotypes and are labeled with αSMA, but their negative immunoreactivity with RPE65 antibody did not support RPEs as their origin. Consistent with previous findings, αSMA-positive RPE-like cells were detected at the lesion sites (Fig. S2C) (He and Marneros, 2013; Strittmatter et al., 2016), but they were not GFP positive.

Our data suggest that infiltrating GFP-positive pericytes maintain expression of perivascular marker PDGFRβ (Fig. 4, Table 1; Fig. S2). Molecular patterns that determine pericyte identity are rather heterogeneous and no single pericyte-specific marker has been identified thus far. Moreover, molecular markers expressed by pericytes are rather dynamic, depending on the developmental or pathological states (Armulik et al., 2011). Previous reports using the same mouse line showed that GFP-positive pericytes in the kidney express myofibroblast marker αSMA after disease induction and contribute to kidney fibrosis (Lin et al., 2008), while in spinal cord, GFP-positive pericytes contribute to fibrotic scars without αSMA expression (Soderblom et al., 2013). In our study, we showed that GFP-positive pericytes in the choroid did not express αSMA before injury (Fig. 1B, Table 1), while infiltrating GFP-positive pericytes were mostly αSMA positive (Table 1; Fig. S2C). These findings indicate that GFP expression might mark different types of cells depending on the tissues, or that GFP-positive pericytes respond to insult in a context-dependent manner.

Pericytes provide structural and functional support to vascular endothelium under physiological condition, in part through VEGF nourishment (Ribatti et al., 2011). During angiogenesis, vascular ‘tip’ cells recruit pericytes by secretion of platelet-derived growth factor (PDGF) to establish mature vasculature and alleviate its dependence on exogenous VEGF, which is considered a potential cause of tumor resistance to anti-VEGF therapy (Giuliano and Pagès, 2013). Therefore, pericyte activation under disease conditions is considered to promote pathological angiogenesis by promoting maturation of new vessels. Accordingly, blockade of PDGF signaling is effective in suppressing choroidal neovascular growth (Dong et al., 2014; Hou et al., 2010; Kumar et al., 2010; Strittmatter et al., 2016), and synergizes with anti-VEGF agents (Jo et al., 2006). Recently, in clinical trials assessing the therapeutic efficacy of anti-VEGF (Lucentis) and anti-PDGF (Fovista) combination in CNV, patients treated with the combined therapy showed no improvement compared with those treated with anti-VEGF monotherapy. Although details of these studies have not been published, the trial outcomes should be interpreted with caution. The 12-month trials set visual acuity as the primary endpoint measurement, but in this time frame, CNV actively remodels in the presence of anti-VEGF reagents. A clinical trial study has demonstrated the efficacy of Lucentis in inhibition of both vascular and fibrotic lesions with significant improvement of visual acuity within 12 months (Brown et al., 2006). Therefore, the effect of additional anti-PDGF reagents in this scenario could be insignificant, as matured neovascularization has not been established, while evaluation at later time points might prove the benefits of combinational therapy. In support of this idea, a post hoc analysis of Comparison of Age-related Macular Degeneration Treatments Trials (CATT) indicates significant occurrence of CNV-associated scars in response to anti-VEGF monotherapy, when assessed at a 2-year time point (Daniel et al., 2014).

Our data show that choroid-derived PDGFRβ^+^ pericytes infiltrated into the subretinal space early after injury and contributed to ECM deposition. Therefore, it is necessary to interfere with pericyte infiltration early during disease progression in order to prevent the subretinal fibrosis that is associated with profound vision loss. Intervention at late stage might not be effective after extracellular scarring has been established. It has been shown that specifically targeting the PDGFR protein signaling pathway, or ablating proliferating PDGFRβ^+^ cells, is effective in suppressing formation of the subretinal ‘myofibroblastic scaffold’ that is mainly composed of PDGFRβ^+^ cells, but subretinal fibrogenic response has not been evaluated in this scenario (Strittmatter et al., 2016). A key question remains unresolved as to whether choroidal pericytes are activated by PDGFR and/or other signaling pathways in response to photocoagulation. Single-cell analysis and elaborate genetic manipulation of choroidal pericytes would be beneficial to understanding how fibrosis-promoting pericytes are activated and recruited. Elucidation of molecular pathways by which choroidal pericytes activate in response to subretinal lesion would pave significant steps towards identifying therapeutic targets to suppress fibrotic development.

ECM components are synthesized and secreted by multiple cell types, including, but not limited to, fibroblasts and macrophages (Yamauchi et al., 1987). We show that infiltrating GFP-positive pericytes upregulated expression of ECM components and colocalized with fibrotic scars (Fig. 5), pointing to a crucial role of choroid-derived pericytes in subretinal fibrosis. Subretinal fibrosis is the end stage of neovascular AMD patients, resulting in retinal degeneration and permanent vision loss without effective intervention. Currently, no effective treatment is available to resolve subretinal fibrotic scars. Clinical studies have even suggested a positive correlation between anti-VEGF therapy and fibrotic scar development (Daniel et al., 2014; Hwang et al., 2011). Thus, our study suggests that choroid-derived pericytes play crucial roles in subretinal fibrosis and could serve as potential therapeutic targets to halt AMD progression. In view of complexity in the process of subretinal fibrosis, genetic tools for lineage tracing and molecular manipulation are of advantage in defining roles of a specific cell type or signaling pathway. Based on the fact that choroid-derived pericytes as labeled by GFP expression in Col1α1-GFP mice contribute significantly to the subretinal lesion after photocoagulation, this reporter mouse line could be a valuable tool to dissect molecular and cellular mechanisms of choroidal pericyte activation and subretinal fibrosis.

In conclusion, our study has identified choroidal perivascular niche as a novel and significant source of subretinal fibrosis, indicating that choroidal pericytes are potential therapeutic targets for intervention of fibrotic and AMD progression.

## MATERIALS AND METHODS

### Animals and surgery

Col1α1-GFP mice were kindly provided by Dr David Brenner from University of California San Diego (La Jolla, CA, USA) and Dr Shuei-Liong Lin from National Taiwan University (Taipei City, Taiwan). The Col1α1-GFP transgenic mice were generated as described (Krempen et al., 1999; Lin et al., 2008; Yata et al., 2003). Briefly, part of the collagen 1 (α1) promoter (−3122 to +111) and enhancer (−8000 to −7000) sequences were cloned and positioned at 5′ of the open reading frame of enhanced GFP (EGFP). GFP expression is driven by the truncated Col1α1 promoter. Animal procedures conformed with the National Institutes of Health (USA) guide for the care and use of laboratory animals (NIH Publications No. 8023, revised 1978), and were approved by the Shanghai Jiao Tong University Institutional Review Board. Mice were subjected to laser-induced photocoagulation as previously described (Apte et al., 2006), with modifications. Briefly, mice (8-10 weeks, six to eight per group) were anesthetized with sodium pentobarbital and the pupils were dilated with 1% tropicamide (Santen, Osaka, Japan). The four laser-injured spots were induced by a 532-nm laser with a power of 150 mW (Visulas 532S; Carl Zeiss Meditec, Dublin, Ireland) in a standard fashion around the optic nerve using a slip lamp delivery system. Anesthetized animals were allowed to recover on a heat pad until sacrifice.

### Histology

Mice were perfused transcardially with 4% paraformaldehyde. Eyeballs were harvested and prepared for immunohistochemistry. Briefly, RPE-choroid complexes were dissected out of eyeballs for flat-mounted analysis or cryosectioned (12 μm). Antibodies or reagents used for histological staining were anti-PECAM1 (550274, BD Biosciences), anti-Glut1 (07-1401, Millipore), anti-PDGFRβ (32570, Abcam), anti-NG2 (AB5320, Millipore), anti-αSMA (5694, Abcam), anti-CD11b (RM2800, Invitrogen), anti-Iba1 (019-19741, Wako), anti-RPE65 (78036, Abcam), anti-vimentin (32322, Santa Cruz Biotechnology), anti-CD13 (33489, Abcam), anti-CD45 (14-0451-81, eBioscience), anti-GFAP (33673, Santa Cruz Biotechnology), anti-glutamine synthetase (73593, Abcam), anti-fibronectin (AB2033, Millipore), anti-collagen IV (19808, Abcam), anti-glutamine synthase (73593, Abcam), anti-caspase 3 (559565, BD Biosciences), anti-Ki67 (1667, Abcam) and DyLight594-labeled anti-isolectin B4 (FL-1207, Vector Laboratories) (all 1:500).

### Isolation of GFP-positive cells with flow cytometry

After laser-induced photocoagulation, the eyecups of mice were gently isolated and subjected to enzymatic digestion with 0.25% trypsin (Gibco) for 20 min at 37°C. The treated tissues were then transferred to Dulbecco's modified Eagle medium (DMEM) (Gibco) supplemented with 5% fetal bovine serum (Gibco). The inner surface of digested eyecups were carefully scraped to dissociate cells at the injured sites. The dissociated cells were then concentrated in DMEM and kept on ice until analysis. Isolation of GFP^+^ cells was carried out on a Beckman Coulter MOFLO XDP Cell Sorter. Fluorescent gates were determined according to background staining displayed by suspended cells from wild-type mice. At least 5000 cells were collected for further analysis.

### RNA extraction and quantitative PCR

Total RNA was extracted from isolated cells with an RNAprep Kit (#DP430, Tiangen Biotech, Beijing, China). cDNA was synthesized with a PrimeScript RT reagent Kit (RR047Q, Takara, Shiga, Japan) and analyzed by real-time PCR (Applied Biosystems, Foster City, CA, USA) according to the manufacturer's instructions. ΔΔCt method was used to obtain the fold change in mRNA expression level. Primer sequences were as follows: *Gapdh* forward: 5′-GACCTCAACTACATGGTCTACA-3′, *Gapdh* reverse: 5′-ACTCCACGACATACTCAGCAC-3′, collagen 1 forward: 5′-AGACGGGAGTTTCTCCTCGG-3′, collagen 1 reverse: 5′-GGGACCCTTAGGCCATTGTG-3′, fibronectin forward: 5′-AACGTGCTATGACGATGGGAA-3′, fibronectin reverse: 5′-AGGGTGGGGCTGGAAAGATTA-3′.

### Quantifications and statistics

Six to eight mice were included as an experimental group, and were injured in the left eye by four laser spots. Samples of the laser-injured spots were imaged with a Leica SP8 confocal microscope (40×), and colocalization was determined using LASX software (Leica) to examine optical slices. Cell counts were performed unbiasedly with immunostained flat mounts or five consecutive tissue sections around the lesion epicenter. One-way ANOVA with Tukey's post test was used to determine statistical significance.

## Supplementary Material

Supplementary information
