# Supplementary material for: Choroidal pericytes promote subretinal fibrosis after experimental photocoagulation
Source: Dis Model Mech. 2018 Apr 23;11(4):dmm032060. doi: 10.1242/dmm.032060 (PMC5963858; doi:10.1242/dmm.032060)
Supplement: Supplementary information [file dmm-11-032060-s1.pdf]

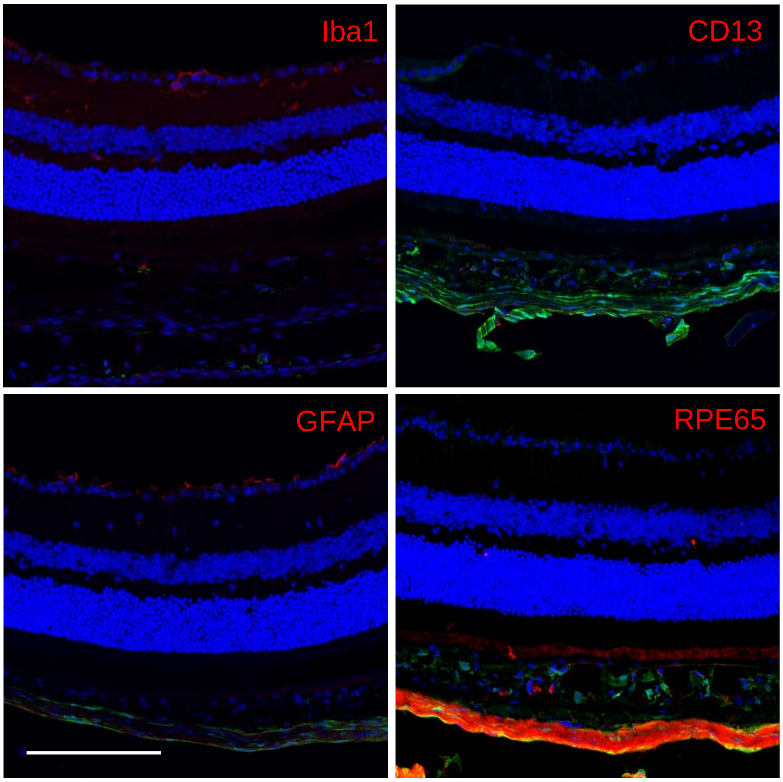

Figure S1. Representative images show cross sections of posterior eye isolated from Col1α1-GFP mice. Samples were immunostained with antibodies against Iba1, CD13, GFAP and RPE65 (n=5). All samples were counter-stained with DAPI (blue), GFP antibodies (green) and the third antibody (red) as shown. Scale bars: 100μm.

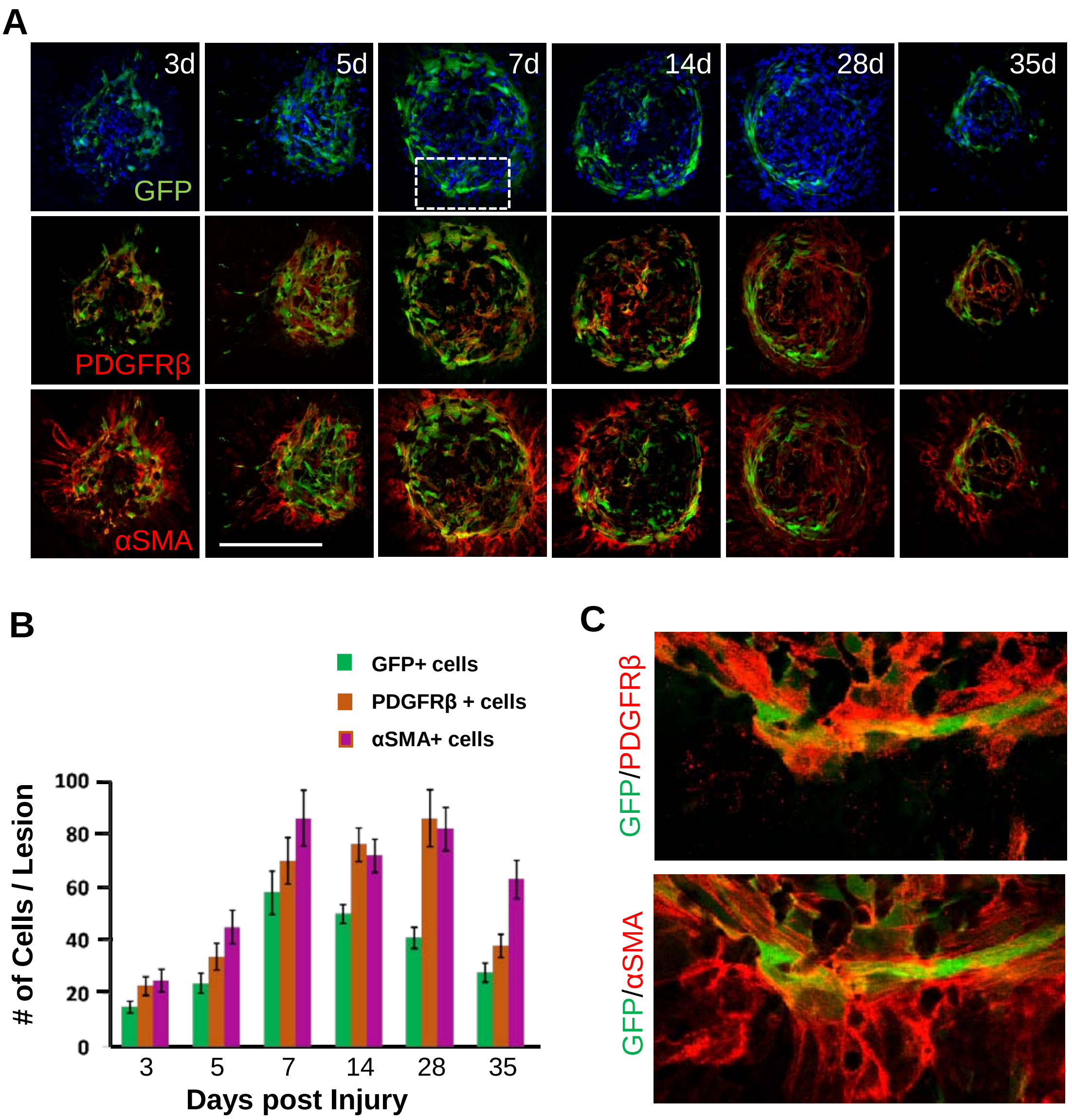

Figure S2. GFP-positive cells express PDGFRβ and αSMA. A, B: The time course of GFP, PDGFRβ and αSMA-positive cell distribution in subretinal space after laser-induced photocoagulation (n>10). C: GFP-positive cells are immunoreactive with antibodies against PDGFRβ and αSMA. Scale bars: 100µm.

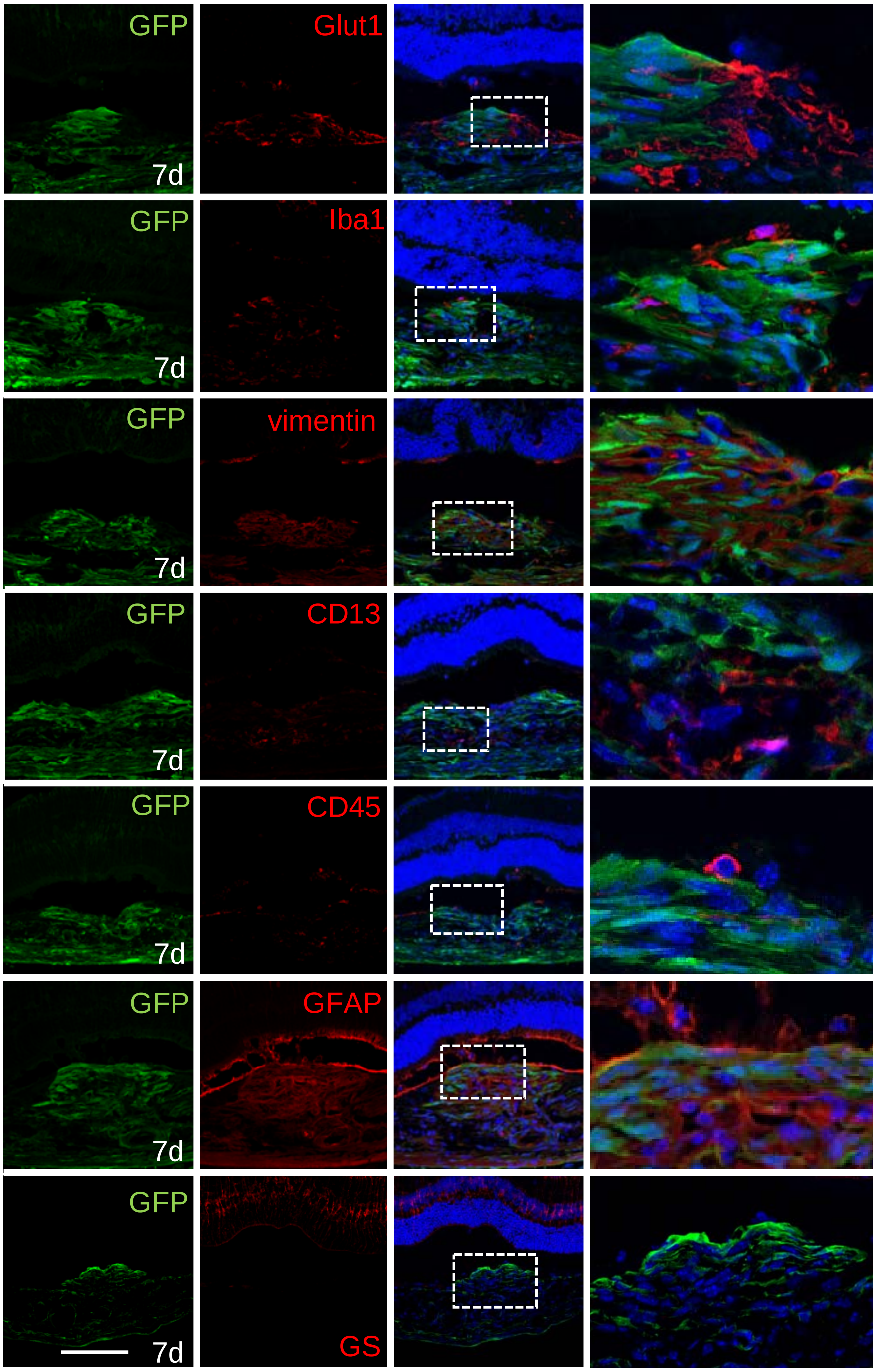

Figure S3. Representative images show cross sections of posterior eye isolated from Col1α1-GFP mice at 7 dpi. Samples with laser-induced lesions were immunostained with antibodies against Glut1, Iba1, vimentin , CD13, CD45, GFAP or glutamine synthetase (GS). All samples were counter-stained with DAPI (blue), GFP antibodies (green) and the third antibody (red) as shown (n=5). Scale bars: 100µm.

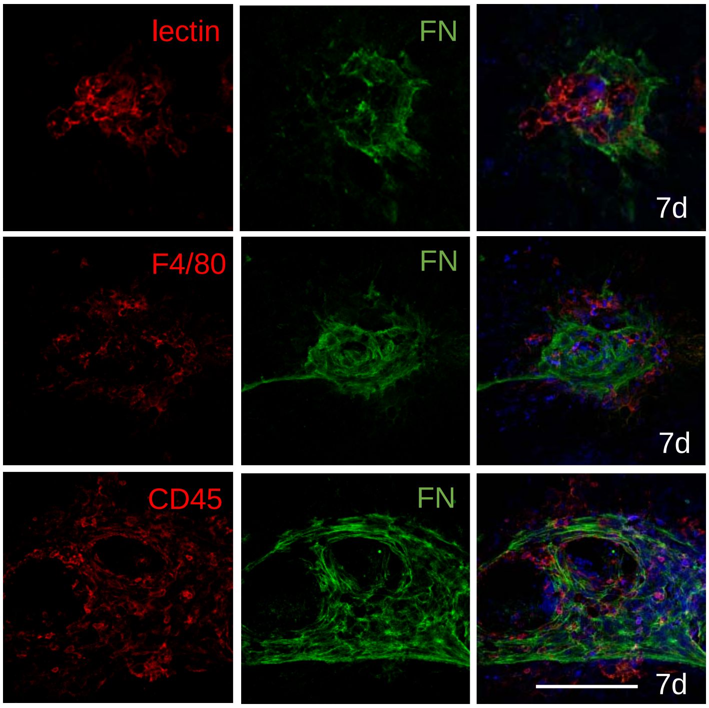

Figure S4. Representative images show flat-mounted RPE-choroid complexes isolated from wild type mice at 7 dpi. Samples with laser-induced lesions were immunostained with antibodies against fibronectins (FN), F4/80, CD45 or lectins. All samples were counter-stained with DAPI (blue) (n=5). Scale bars: 100μm.
